# Estimation of Proinflammatory Factors in the Saliva of Adult Patients with Cystic Fibrosis and Dental Caries

**DOI:** 10.3390/medicina56110612

**Published:** 2020-11-14

**Authors:** Tomasz Hildebrandt, Anna Zawilska, Agata Trzcionka, Marta Tanasiewicz, Henryk Mazurek, Elżbieta Świętochowska

**Affiliations:** 1 Department of Conservative Dentistry with Endodontics, Faculty of Medical Sciences in Zabrze, Medical University of Silesia, Plac Akademicki 17, 41-902 Bytom, Poland; thildebrandt@sum.edu.pl (T.H.); azawilska@sum.edu.pl (A.Z.); martatanasiewicz@sum.deu.pl (M.T.); 2 Department of Pneumonology and Cystic Fibrosis, Institute of Tuberculosis and Lung Disorders, ul. Prof. Jana Rudnika 3B, 34-700 Rabka Zdrój, Poland; hmazurek@igrabka.edu.pl; 3 Department of Medical and Molecular Biology, Faculty of Medical Sciences in Zabrze, Medical University of Silesia, ul. Jordana 19, 41-808 Zabrze- Rokitnica, Poland; elaswieta@interia.pl

**Keywords:** cystic fibrosis, tooth decay, resistin, lysozyme, lactoferrin, α-amylase activity, salivary pH and buffer capacity

## Abstract

*Introduction*: The available literature lacks data regarding the levels of resistin, lysozyme, lactoferrin, α-amylase activity, pH, and saliva buffer capacity, as well as oral health and hygiene in the group of adult patients with cystic fibrosis (CF). The aim of the research was to assess the selected saliva parameters in patients diagnosed with cystic fibrosis. *Materials and methods*: Examined group was composed of 40 patients diagnosed with CF, while the control group of 40 healthy individuals. Both groups underwent the same scheme of the assessment (DMT index, salivary pH, buffer capacity, analysis of total sialic acid, total protein estimation, lysozyme levels estimation, lactofferin levels measurement, α-amylase activity, estimation of the levels of resistin and TNF-α). *Results*: In the examined group, there were higher values of decayed teeth as well as values of sialic acid, total protein, lactoferrin, α-amylase, and TNF-α. However, mean lysozyme, and resistin levels, as well as pH and buffer capacity of the saliva, were lower. *Conclusions*: New diagnostic methods, including the evaluation of selected salivary biochemical parameters, may indicate the existence of factors predisposing to severe tooth decay in the study group. Appropriate preventive treatment to combat dental caries in adult patients with CF will significantly improve their comfort and life expectancy.

## 1. Introduction

Cystic fibrosis (CF) is an autosomal recessive disorder found mainly in Caucasians. According to various sources, the estimated incidence of CF is between 1:2000 to 1:2500 worldwide, and 1:5138 in Poland [1].

Salivary pH and composition have significant effects on oral health in CF patients. Studies have shown a reduced volume of salivary production due to impaired exocrine secretion or secondary to damaged salivary glands. Carbohydrates are one of the main etiological factors of tooth decay. CF patients have higher energy demand than healthy individuals, which requires increased consumption of high-energy cariogenic food products or dietary supplements, which, in turn, promotes dental caries development and increases its severity [2,3].

A decreased volume of secreted saliva, reduced pH, and salivary buffer capacity are cariogenic factors. An evaluation of total salivary proteins, albumins, and mucins allows for determining the level of enamel protection against cariogenic bacteria [4]. Furthermore, the measurement of salivary levels of soluble TNF-α and β2-microglobulin was shown to be useful in the assessment of chronic inflammatory disease [5]. Chronic inflammation in CF is often associated with a negative energy balance and the loss of body cell mass. These changes result from the action of proinflammatory cytokines such as tumour necrosis factor (TNF), interleukin-1 (IL-1), and IL-6, all of which are elevated in active inflammation [4].

Saliva is one of the biological secretions in humans. It is produced regularly, and its composition is similar to that of blood or urine. Therefore, it seems that its role in diagnostic laboratory investigations is still underestimated.

The available literature lacks data regarding the levels of resistin, lysozyme, lactoferrin, α-amylase activity, pH, and saliva buffer capacity, as well as oral health and hygiene in the group of adult patients with CF. The latest literature is limited only to studies in children. Therefore, the proposed subject matter and scope of research may be a significant contribution to current knowledge on oral health in adult patients with CF. The aim of the work was an assessment of selected salivary parameters in CF patients based on:

- physicochemical indicators: pH and salivary buffer capacity
- biochemical indicators: total sialic acid as a marker for mucins, total protein, lysozyme, lactoferrin, α-amylase activity, and resistin and TNF-α levels.

## 2. Materials and Methods

A total of 40 adult patients (over 18 years of age) with diagnosed and genetically confirmed CF were included in the study. These patients were hospitalised in the Institute of Tuberculosis and Lung Diseases, Department of Pulmonology and Cystic Fibrosis in Rabka-Zdrój due to disease exacerbation.

The control group included 40 randomly selected healthy individuals age- and sex-matched to the study group. These were patients of the General Dentistry Clinic at the Academic Centre of Dentistry and Specialist Medicine in Bytom, who, for the first time, reported to the facility for dental treatment.

Inclusion criteria: Patients over 18 years old with clinical and genetic diagnosis of CF.

Exclusion criteria: Patients diagnosed with other, severe general diseases, autoimmunological diseases, chronic kidney disease, diabetes, neurological or psychiatric diseases, after lungs transplantation, treated with immunosuppressive drugs or sex hormone drugs, dysfunction of the masticatory system.

The project of the research obtained agreement of Bioethical Commission of Medical University of Silesia in Katowice (resolution no. KNW/0022/KB1/172/12/13, approved: 22.01.2013).

### 2.1. Examination of Patients

The examination of patients hospitalised in the Institute of Tuberculosis and Lung Diseases, Department of Pulmonology and Cystic Fibrosis in Rabka-Zdrój was performed in treatment rooms under artificial lighting (headlamp). The controls were examined in the General Dentistry Clinic at the Academic Centre of Dentistry and Specialist Medicine in Bytom on a dental chair, under artificial lighting (the unit’s lamp). The oral cavity status assessment was performed by one operator, who was trained before he started the procedure. He was supervised by two experienced, trained researchers.

A standardised diagnostic kit (mouth mirror, probe, dental tweezers) and a calibrated WHO-621 periodontal probe were used. Dental status, oral hygiene, and periodontal therapeutic needs were assessed. The obtained clinical findings were recorded.

### 2.2. Oral Health Assessment

Dental health was assessed for decayed, missing and filled teeth described as the DMFT indicator (D—decayed, M—missing, F—filled), and the mean DMFT for both the CF and the control group. Due to significant variability in the prevalence of third molars (impacted teeth, teeth removed for orthodontic reasons), these were not considered in the measurement of DMFT [1]. Dental Treatment Index (DTI) was also used in the study. It is calculated by dividing the number of filled teeth (F) by the total number of filled and decayed teeth (F+D). DTI may reflect dental treatment in one patient, a group or a large population of patients. The index ranges from 0 to 1. Values in the range of 0.0–0.5 indicate the lack of or poor efficacy of treatment, while a range of 0.6–1.0 indicates high efficacy [6].

### 2.3. Salivary Test

Salivary samples were collected in the morning hours (between 8 and 11 a.m.). Before sampling, patients in the CF and the control group refrained from eating, brushing their teeth, smoking cigarettes, chewing gum, and consuming fluids, if possible, considering the general condition of the patient and the degree of dryness in the oral cavity.

### 2.4. Salivary pH Measurement

A potentiometric method with a glass electrode (ORION 9609BN, Waltham, MA USA), allowing for selective measurement of H^+^ ions and a pH-meter (model 370, ORION, Waltham, MA, USA) were used.

### 2.5. Determination of Salivary Buffer Capacity

The measurement was performed using a colorimetric chemical test known as CRT Buffer (Ivoclar—Vivadent). A drop of saliva was placed on the test strip. After 5 min, on the basics of the colour obtained on the strip, the buffer capacity was defined as: low (yellow, pH ≤ 4.0), medium (green, pH 4.5–5.5), high (blue, pH ≥ 6).

### 2.6. Analysis of Total Sialic Acid

Spectrophotometric method according to Jourdian G.W. et al. was used for this measurement [7].

Sialic acid was analysed in saliva centrifuged for 10 min at 13,000× *g* rpm at room temperature. A periodate-resorcinol method was used for the quantitative estimation of total, bound (via a glycosidic linkage) and free sialic acid. This method is based on periodate oxidation of sialic acid to form a chromogen, which reacts with resorcinol to produce a coloured compound. Colour intensity was measured spectrophotometrically at 630 nm against the blank. The levels of sialic acid were read from a standard curve for 100 μg/mL N-acetylneuraminic acid solution (Fuka, Switzerland).

The level of sialic acid was expressed in mg/L. The precision of the method in a simultaneous series (imprecision of the method) was 4.8%, and the reproducibility was 7.3%.

### 2.7. Total Protein Estimation by Lowry’s Method

Total salivary protein was estimated by a method using reactions that occur between peptide bonds and tyrosine and the Folin–Ciocalteu reagent [7]. The obtained results were expressed in mg/mL. Imprecision of the method (repeatability in a simultaneous series) was 2.5%.

### 2.8. Estimation of Lysozyme Levels

A two-step immunoenzymatic method based on the ELISA test (Innovative Research Inc., Novi, MI USA) was used for the measurement of lysozyme levels [8]. The reading was performed at 405 nm, 30 min after adding the dye, using the µQuant reagent (Bio Tek, Winooski, VT, USA), while the processing of results was performed using the KCJunior (BioTek, Winooski, VT, USA). Human milk lysozyme was used as standard (Sigma-Aldrich). The obtained results were expressed in μg/mL. Imprecision of the method was 4.1%.

### 2.9. Estimation of Lactoferrin Levels

The levels of lactoferrin were estimated using a similar approach to the one used for lysozyme, using a two-step immunoenzymatic method based on the ELISA test (Calbiochem, San Diego, CA, USA) [8]. Absorbance was recorded using the µQuant reader (Bio Tek, Winooski, VT, USA), while the processing of results was performed using the KCJunior (Bio Tek, Winooski, VT, USA). The obtained results were expressed in μg/mL. Imprecision of the method was 4.8%.

### 2.10. Measurement of α-Amylase Activity

A static method [9] using a kit (Aqua-Med., Łódź, Poland) was used for the measurement of α-amylase activity; 2-chloro-4-nitrophenylmaletrioside is a substrate in this method, and the reaction was performed in the MES buffer at pH of 6.9 and 37 °C. The spectrophotometric reading of the resulting coloured product was performed at 405 nm. Saliva samples were diluted 100 times with 0.9% NaCl. The obtained results were expressed in the units of salivary α-amylase activity (U/mL). Imprecision of the method was 4.1%.

### 2.11. Estimation of the Levels of Resistin and TNF-α

Commercially available ELISA (Bender MedSystems GmBh, Vienna, Austria) tests were used for the estimation of the levels of resistin and TNF-α [8]. The analytical procedure complied with the manufacturer’s technological instructions included in the kits. Absorbance readings were performed using the µQuant reader (Bio Tek, Winooski, VT, USA), while the processing of results was performed using the KCJunior (Bio Tek, Winooski, VT, USA). The sensitivity of the method was 0.2 ng/mL for resistin and 0.1 pg/mL for TNF-α. Imprecision of the method was 5.2% and 4.9% for resistin and TNF-α, respectively.

### 2.12. Statistical Methods

Physical examination and medical history data were compiled in Microsoft Excel spreadsheet and analysed statistically. In order to answer the research questions, statistical analyses were conducted using the IBM SPSS Statistics 23. Using the Package, an analysis of basic descriptive statistics and a number of Mann-Whitney tests, chi-square independence test, as well as Spearman correlation coefficient analyses were performed. A *p* < 0.05 was considered statistically significant. Results at 0.05 < *p* < 0.1 were considered statistically significant at the level of statistical tendency.

## 3. Results

### 3.1. Characteristics of the Study Group

The CF group included 40 adult patients diagnosed with CF between age of 18 to 39 years (mean age 26 ± 6.46 years), including 24 women (60%) and 16 men (40%), treated in the Institute of Tuberculosis and Lung Diseases in Rabka Zdrój. The control group included 40 adult patients aged between 18 and 37 years (mean age 24.33 ± 5.11), including 25 women (62.5%) and 15 men (37.5%) treated in the General Dentistry Clinic at the Academic Centre of Dentistry and Specialist Medicine in Bytom (Table 1).

### 3.2. Oral Status

DMFT and mean DMFT were estimated in both groups to assess oral health in every patient. Based on the DMFT, dental treatment index (DTI) was calculated.

The analysis of oral health based on the mean DMFT showed higher values in the CF vs. control group (14.62 vs. 11.15; *p* = 0.004). The measure of the effect size of differences (r) showed that these were medium effect sizes.

Significant differences were found for only one component of DMFT. Decayed teeth (D) were reported more often in the CF group vs. controls (4.28 vs. 2.98; *p* = 0.048).

No significant differences were found in the mean DTI or the severity of cries between the groups (Table 2).

### 3.3. Biochemical and Physicochemical Parameters

A comparative analysis was performed for biochemical and physicochemical parameters estimated for stimulated saliva of CF patients and controls. Mean levels of sialic acid, total protein, lactoferrin, salivary α-amylase, and TNF-α were significantly (*p* < 0.001) higher in CF patients vs. controls.

Mean lysozyme and resistin levels were significantly lower (*p* < 0.001) in CF patients vs. controls.

For the purpose of statistical analysis of the pH of saliva samples in the CF and control group, the obtained values were converted into ion levels [H^+^], as in accordance with the definition of pH, and then mean [H^+^] levels were calculated for both groups. The calculated values were subsequently converted into mean pH values for each group.

Significantly lower mean pH values were reported for CF patients (6.28; *p* = 0.012 vs. 7.05 *p* = 0.012).

The analysis of salivary buffer capacity in both groups showed significantly lower values in CF patients (6.04 ± 0.52 mL; range: 4.9 to 6.8) compared to controls (6.73 ± 0.2 mL range: 6.4 to 7.1) (Table 3.).

### 3.4. The Relationship between the Levels of Selected Salivary Parameters and Oral Health Indices

Spearman’s rho was used to assess the relationship between oral health indices and the selected salivary biochemical parameters.

No tendency or significant correlation between the levels of selected physicochemical and biochemical parameters of saliva and oral health indices were found in the CF group.

The statistical analysis of oral health data in the control group showed a significant relationship between the levels of lactoferrin (W *r* = −0.351; *p* = 0.026 DMF *r* = −0.399; *p* = 0.011) and α-amylase activity (W *r* = 0.349; *p* = 0.027 DMF *r* = 0.390 *p* = 0.013) and the number of filled teeth (F) and the total number of decayed, filled and missing teeth (DMF). The correlation with the levels of lactoferrin was moderately strong and negative. The activity of α-amylase showed a moderately strong and positive correlation. In the control group, a significant correlation was found between pH (*r* = −0.342; *p* = 0.031), salivary buffer capacity (*r* = −0.326; *p* = 0.040), total protein (*r* = −0.393; *p* = 0.012) and the DMF index. These were strong negative correlations (Table 4).

## 4. Discussion

The available literature on oral health in patients with CF is devoted mainly to the paediatric population. In the present study, the mean age of CF patients was 26 years, with the youngest patient aged 18 years and the oldest patient aged 39 years. Therefore, this adult population may be an important continuation and contribution to the knowledge on the effects of proinflammatory factors on tooth decay in this group of patients.

Oral health in CF patients is determined by 4 major factors: 20 times higher count of *Streptococcus mutans*, gastroesophageal reflux disease (GERD), high-calorie diet, and enamel damage [10,11]. Despite such significant risk factors, the prevalence of dental caries seems lower in CF patients, as pointed out by many authors [12,13,14,15]. However, most of these studies were conducted in a paediatric population, with adults accounting for only a small group of patients. According to some authors, CF patients present with a higher tendency to develop tooth decay [16], while other authors report no significant differences in this regard [17,18]. These discrepancies may result e.g., from the choice of control group, for which the following aspects were considered: division into age groups, other respiratory diseases, family history of the disease, and demographic data. The mode of examining patients and non-harmonised research standards are other important factors affecting the obtained results. The training and experience of the investigator, as well as the conditions in which the research was conducted, are also important. Our study showed higher susceptibility to caries in adult CF patients, as confirmed by an analysis conducted by Dąbrowska et al. [16].

Protective factors that prevent dental caries include, among other things, salivary pH and the associated buffer capacity.

The mean pH values in our study were significantly lower in the CF group compared to controls (6.28 vs. 7.05), which was also confirmed by Gonçalves et al. [19]. Catalán et al. [10] found in a murine model that lower pH causes dental plaque acidification and, consequently, carious lesions.

Alkhateeb et al. [20] showed mean pH of 7.14 ± 0.48, which was higher than in our study. Sui et al. [21] demonstrated that children with CF had significantly reduced salivary pH.

Inhaled drugs used by patients with CF also have an important impact on salivary pH in this population. Tootla et al. [22] showed reduced salivary pH in asthmatic patients after the use of inhaled corticosteroids or β2-agonists, which may suggest a similar correlation in CF patients.

Salivary buffer capacity largely depends on bicarbonate and phosphate buffer, as well as salivary proteins [23]. Some authors showed that CF affects ion metabolism (chlorides and sodium bicarbonate). According to Rigas et al. [24], patients show reduced activity of phosphate buffer with unchanged activity of bicarbonate buffer. Kinirons [13] found that reduced prevalence of dental caries in patients with CF was associated with mean salivary pH and significantly increased stimulated salivary buffer capacity, which corresponds with our study in adult patients. Alkhateeb et al. [20] observed that 26% of patients with CF had high salivary buffer capacity (final pH > 4.75), 26% had mean salivary buffer capacity (final pH = 4.25–4.75), and 48.1% had low buffer capacity (final pH < 4.25). However, direct comparison of these studies is not possible as they were conducted among children.

Da Silva Modesto et al. [25] also found no differences in pH and salivary buffer capacity between the analysed groups of CF children.

Our study showed no correlation between salivary physicochemical indices and oral health indices in adult patients with CF. 

According to the available literature on inorganic salivary components in CF patients, there are increased levels of proteins and calcium, which allows for the formation of insoluble calcium–protein complexes that adversely affect salivary enzymes [26].

Also, CF patients were reported to have increased salivary levels of sodium, potassium, phosphate, and chlorides [19,24]. The above-mentioned studies focused on inorganic salivary components. However, only a limited number of papers focus on organic components of saliva, including its antioxidant and antibacterial activity, especially in the group of adult patients with CF. Mucin glycoproteins (salivary mucins) belong to a group of compounds that protect the teeth and the oral mucosa against harmful effects of proteases and enzymes produced by bacteria. High-molecular-weight mucins (MG1), which are found in the oral cavity, and low-molecular-weight mucins (MG2), are present in the human GI tract e.g., the oesophagus. Mucins ensure direct dental protection against caries by an aggregation followed by an elimination of *Streptococcus* strains from the oral cavity. As the main component of acquired pellicle (which forms immediately after brushing the teeth), they interact with hard dental tissue, thereby contributing to oral microbiological composition [27]. The protective effects of mucins were confirmed by Baughan et al. [28], who showed a correlation between increased salivary *S. mutans* titres and reduced levels of mucins. Furthermore, they form a thin layer acting as a lubricant, thereby protecting oral surfaces against mechanical factors causing their damage or abrasion.

Sialic acid (N-acetylneuraminic acid) is a component of the sugar moiety of mucins. Since the content of sialic acid is positively correlated with the levels of mucins, we assumed in our study that salivary levels of sialic acid were an indicator of mucin production. In medical diagnosis, sialic levels are measured in patients with systemic diseases, including CF. Its high salivary levels were reported for cancer, cardiovascular diseases, rheumatoid arthritis, and inflammatory reactions. It is also a biomarker of myocardial infarction and diabetes [29]. Both free and bound sialic acid is found in saliva and blood. As a strong acid at normal salivary pH, it is completely ionised, which could contribute to tooth decay. Nevertheless, the study showed no direct effects on caries intensity. However, an ability of sialic acid contained in salivary mucins to aggregate *Streptococcus sanguis* was observed [30]. Rathod et al. [31] showed a significant relationship between increased levels of sialic acid in oral and gingival epithelial cells and periodontal diseases. The authors attribute this relationship to the increased enzymatic activity accompanying periodontal inflammatory disease, including neuramidase.

Our study showed significantly higher mean total sialic acid in CF patients (65.2 ± 6.42) compared to controls (42.7 ± 4.45), suggesting that salivary sialic acid may also be a marker for an ongoing inflammatory process. Naturally, this requires further research in a larger group of patients. On the other hand, Da Silva Modesto et al. [25] showed lower salivary levels of sialic acid in paediatric patients with CF, which was probably associated with age-dependent disease onset and severity. When interpreting these studies, attention should be paid to the drugs used by patients, including mucolytic agents, which may affect the production of salivary glycoproteins and reduce salivary flow rate, thus posing a risk to oral health, particularly to the oral mucosa. Our study showed no tendency or significant correlations between the levels of sialic acid and oral health/oral hygiene indices in the group of adult CF patients. However, a significant positive intercorrelation was observed between the levels of sialic acid and total protein, which is due to the fact that inflammation induces increased involvement of specific proteins in this process, which consequently leads to increased salivary total proteins.

Helmerhorst and Oppenheim [32] reported 309 proteins in the whole saliva. Proline-rich acidic and basic proteins, amylases, high and low molecular weight salivary glycoproteins, agglutinins, cystatin, histidines, and stearins accounted for more than 95%.

Salivary proteins are involved in many biological processes, such as maintaining tissue integrity, oral pH regulation, or antibacterial activity. Their levels may depend on the circadian rhythm, hormones, mental disorders, or oral hygiene [33]. Aps and Martens [34], who investigated salivary levels of total protein in CF patients showed (statistically insignificantly) increased salivary total protein in homozygous patients compared to heterozygous patients and controls. The authors have speculated that there is an internal compensatory mechanism for salivary protein levels. However, this needs to be thoroughly investigated. Other authors also reported increased salivary proteins in CF patients, which may be associated with altered integrity of oral and gingival mucosa, and thus elevated salivary albumins [35]. Our study in adult CF patients confirmed the increased salivary proteins compared to controls. Exacerbation of systemic inflammation in CF causes an increase in the levels of specific inflammatory proteins in saliva. This was confirmed by Nie et al. [36], who showed elevated levels of proteins such as VEGF, IP-10, IL-8, and EGF, as well as lower levels of MMP-9. It should also be noted that in addition to inflammatory markers, other proteins involved in maintaining the integrity of dental structures, maintaining proper balance between demineralisation and remineralisation, in particular, are found in saliva [37].

Tulunoglu et al. [38] showed active dental caries in patients with elevated total salivary proteins. These findings are confirmed by the latest research by Pandey et al. [39]. The correlation between caries intensity and total salivary proteins may have both positive and negative impact on tooth decay. Proteins responsible for maintaining normal oral pH by interacting with the salivary buffer system, which show bactericidal properties play a protective role, whereas adhesins and agglutinins contribute to bacterial colonisation of the oral cavity.

Elevated salivary proteins are also important biomarkers for gingival and periodontal diseases, and are associated with their leakage from plasma. Shaila et al. [40] confirmed this relationship. Aps et al. [19] showed that homozygous CF patients present with significantly reduced tartar, as confirmed in our study. It should be noted that reduced subgingival tartar reduces the risk of periodontal diseases, but at the same time, its presence provides a barrier preventing the release of plasma proteins in coexisting inflammation, as suggested by Banderas-Tarabay et al. [41]. Reduced salivary production is observed in CF patients [19,20,25], which may contribute to increased total protein. According to Sánchez et al. [42], increased levels of mucins, amylase, and total protein may result from reduced salivary flow. The author also points to the mechanism regulating salivary production, which is mediated by the sympathetic and parasympathetic nervous systems. The presence of dental plaque and inflammation activates salivary production via neural transmission. Therefore, it seems possible that periodontal inflammation causing tissue damage activates the sympathetic system and induces an increased secretion rate of certain proteins, thereby enhancing the salivary protective potential. This is confirmed by the fact that total protein and amylase levels return to standard levels once the treatment of gingival and periodontal inflammation is completed [43]. The above-discussed phenomenon may also be observed in adult CF patients, which was partly confirmed in our study. Therefore, future research should be conducted in a larger study population, including patients with severe periodontal and gingival inflammation.

No tendency or significant correlations between total salivary protein and oral health/hygiene indices were found in the CF group. However, a significant correlation was observed between total protein levels and caries intensity, which may confirm the protective function of salivary proteins. A correlation at the level of statistical tendency between protein levels and the approximal plaque index was reported in the same group.

Lysozyme belongs to proteins of particular bactericidal importance [44]. Hughes et al. [45] showed significantly increased plasma and salivary lysozyme levels in their study in a group of CF patients aged between 5 and 33 years. There are documented reasons for this phenomenon. The first reason is a secondary increase in neutrophil counts due to chronic bacterial respiratory infection. The second one, resulting from the underlying disease, is a defect of the lysosomal membrane, which allows for increased hydration of this enzyme. Despite high lysozyme levels, there is no evidence for its bactericidal effects on the basic pathogens in CF, such as *Staphylococcus aureus* and *Pseudomonas aeruginosa*. Hughes et al. [45] also pointed to fluctuating lysozyme levels, which are not constant, and suggested their relationship with exacerbated inflammation.

Our studies showed a significant increase in the levels of lysozyme in adult CF patients, which confirms previous reports. As for dental caries, Iacono et al. [46] showed its bactericidal effects on *S. mutans*. Although high oral levels of lysozyme should suggest a decreased prevalence of tooth decay, the data reported in the available literature is ambiguous. Jentsch et al. [47] and Felizardo et al. [48] observed higher susceptibility to caries among adult patients despite increased salivary levels of lactoferrin and lysozyme, whereas Mass et al. [49] reported an inverse relationship, which may indirectly account for the protective role of proteins in the aetiology of dental caries. Our study in adult CF patients showed no significant correlation between DMF-T and oral hygiene indices and lysozyme levels despite its high salivary levels. A significant negative intercorrelation between lysozyme levels and lactoferrin was only observed in the control group.

These contradictory literature reports and our own findings confirm the need for further research on the role of lysozyme in the aetiology of tooth decay, with particular emphasis on adult patients with CF.

Lactoferrin also exhibits antibacterial properties. It is one of at least 45 different antimicrobial proteins found in saliva [50].

There are no reports on salivary levels of lactoferrin in adult CF patients in the available literature. However, Caraher et al. [50] emphasised in their studies that lactoferrin has bactericidal effects on Burkholderia cepacia found in the respiratory tract of CF patients. The same studies also showed that lactoferrin enhances the effects of rifampicin used in the treatment of CF. Rifampicin combined with other antibiotics has bactericidal effects on cariogenic bacteria [51]. Such action of lactoferrin may suggest its potential to reduce caries intensity in patients with CF. However, this needs to be supported by further research taking into account drugs used by patients with CF.

According to Kim et al. [52], lactoferrin shows probiotic activity without inhibiting the effects of *Lactobacillus casei*, but at the same time, it shows no bactericidal effects on *S. mutans*, which is responsible for dental caries.

Glimvall et al. [53] showed that lactoferrin may be a treatment indicator for aggressive and chronic periodontitis. Its increased salivary levels are attributed to increased production of saliva and/or gingival crevice fluid. Its levels drop after effective treatment.

Felizardo et al. [48], who conducted their study in a group of healthy children, found a correlation between the levels of lactoferrin, lysozyme, and caries index. High salivary levels of protein and lysozyme (statistically insignificant) were associated with high DMFT. High lactoferrin levels correlated with high DMFT, the number of filled teeth (F) in particular, were also important. The research indicates that selected salivary proteins may be used for the assessment of the risk of dental caries in the future.

Our study demonstrated significantly increased salivary levels of lactoferrin in adult patients with CF. However, no correlation was observed between dental caries indices, oral hygiene indices, and the levels of lactoferrin. Significant negative correlations between lactoferrin levels and the F-component of DMFT and mean DMFT were reported in the control group. This confirms literature data on the protective properties of lactoferrin.

According to Burns et al. [54], reduced activity of bactericidal enzymes may have negative effects on oral health in CF patients. The authors reported increased susceptibility to infections caused by *S. aureus*, *Haemophilus influenzae, Burkholderia cepacia,* and *P. aeruginosa* in their study. Recent research on the effects of α-amylase on tooth decay showed its significantly increased levels in patients with active tooth decay and reduced levels in caries-free individuals [55,56]. Single reports on the lack of correlation between α-amylase and caries, as well as papers describing its protective effects on hard dental tissues [57], may be found in the available literature. Direct comparison of these studies is impossible due to different sample sizes, patient’s age, and methodology used for the estimation of salivary α-amylase levels.

Diagnosis of acute respiratory infection in CF patients may pose difficulty due to the possible absence of typical symptoms such as fever, leukocytosis, deteriorated lung function, or the presence of bacteria in the sputum. Therefore, the diagnosis of exacerbations is based on the subjective symptoms associated with health deterioration. Tumour necrosis factor (TNF-α) is a proinflammatory cytokine with pleiotropic effects, which is produced by different types of cells, such as macrophages, white blood cells, epithelial cells, adipocytes, smooth muscle cells, respiratory epithelial cells, and cardiomyocytes [58]. TNF-α also exhibits local effects on immune activation by increasing IL-1 and IL-6 production (it acts as a transcription activator for these cytokines). Furthermore, it enhances both the production and expression of adhesive molecules (ICAM-1, VCAM-1, P- and E-selectins). Studies linking salivary TNF-α levels and oral health pointed to the possible use of this cytokine for, among other things, risk assessment for lichen planus. Estimation of salivary TNF-α seems important for the monitoring of oral inflammation exacerbations and the effects on the oral ecosystem. The analysis of patient’s sputum revealed increased TNF-α in response to a chronic inflammatory process [59].

Studies that do not confirm the significant correlation between TNF-α and exacerbated respiratory inflammation in CF may also be found in the available literature [60]. Increased salivary TNF-α due to inflammation may generate periodontal diseases [61].

Passoja et al. [62] showed high salivary TNF-α levels in patients with advanced periodontal disease. Similar findings were obtained by Frodge et al. [63], who found elevated salivary markers such as β-glucuronidase, CRP, IL-1, MMP-8, and TNF-α, which may suggest their potential use in the diagnosis of periodontal diseases.

Our study points to elevated salivary TNF-α in adult CF patients compared to controls. It should be noted, however, that the selection of CF sample (CF patients) was slightly problematic as salivary samples were collected from patients often hospitalised during disease exacerbation. Therefore, it was impossible to clearly conclude whether the assessed TNF-α levels were not merely evidence for exacerbation. This requires further research that will take into account the drugs used, as well as a thorough evaluation of the ongoing inflammation in patients with CF. The estimation of the levels of TNF-α as an inflammatory marker in periodontal diseases in this group of patients seems of limited value due to the presence of systemic inflammation, as confirmed by other authors [62,63]. Our study showed no correlation between salivary TNF-α and oral health indices in adult CF patients.

The latest publications on inflammatory markers report the potential role of resistin as a systemic and/or local inflammatory indicator. Resistin, which is a cysteine-rich protein, is involved in different biological processes that take place in the human body, including inflammation. It is expressed in monocytes, macrophages, neutrophils, and lymphocytes, as well as the external and internal adipose tissue [64].

The first reports on this subject were presented by Holcomb et al. [65]. Earlier data concerned the relationship between resistin and obesity, type 2 diabetes mellitus, and atherosclerosis. Significantly increased resistin levels were detected in patients with manifestations of infections compared to healthy individuals, but no correlation was observed between its levels and inflammatory markers [66]. Furthermore, Bo et al. [67] hypothesised that resistin is produced in response to moderate chronic inflammation. Hyperresistinaemia in blood and bodily fluids was observed in synovitis of the knee, rheumatoid arthritis, non-alcoholic fatty liver disease, and Crohn’s disease [67,68]. Pontikides et al. [69] showed increased resistin levels in children with allergic rhinitis compared to healthy children. High serum resistin levels were also found in patients with pneumonia and chronic obstructive pulmonary disease. Considering the above, it seems reasonable to estimate salivary resistin in patients with CF. Akram et al. [70], who assessed resistin levels in the gingival crevice, hypothesised that monocytes and macrophages present in chronic periodontal disease may induce increased resistin levels by stimulating proinflammatory cytokines. In this way, the author confirmed that resistin may be used as a marker of chronic periodontitis. However, he did not confirm its significantly increased levels in patients with periodontitis and coexisting systemic inflammation compared to patients with periodontitis only. Our study showed lower resistin levels in CF patients compared to controls, which may be probably explained by the implemented treatment of the underlying disease. All hospitalised patients were in the phase of exacerbation of the disease, which required the inclusion of antibiotic therapy. Perhaps the low resistin levels were due to effective treatment of exacerbated inflammation and/or interaction of drugs with reagents used for resistin estimation. The research should be continued in a larger population of patients, taking into account disease exacerbations and pharmacotherapy used.

No correlation was found between salivary levels of resistin and oral health/oral hygiene indices in this patient population.

## 5. Conclusions

Lower salivary pH, buffer capacity, as well as stimulated and non-stimulated salivary volume in adult patients with CF are factors that promote the spread of dental caries and periodontal and oral mucosa diseases.

Higher salivary levels of total sialic acid, total protein, and TNF-α in CF patients confirm the ongoing inflammation at both the systemic and oral level. On the other hand, increased activity of salivary α-amylase and elevated levels of lactoferrin are indicative of the protective effects of these factors against dental caries.

## Figures and Tables

**Table 1:** Characteristics of the study and control group.

	CF Group (*n* = 40)		Control Group (*n* = 40)		
	M	SD	M	SD	*p*
Age (years)	26.00	6.46	24.33	5.11	0.202
Height (cm)	165.30 *	8.65	169.70	10.38	0.039
Body weight (kg)	56.51 *	12.53	64.20	14.15	0.012
BMI (kg/m^2^)	20.50	3.37	22.10	3.62	0.043
Age at CF diagnosis (years)	7.08	6.20			

Legend: * *p* < 0.05.

**Table 2:** An analysis of mean values of the DMFT components and DTI in the CF and the control group.

	CF Group (*n* = 39)		Control Group (*n* = 40)			
	M	SD	M	SD	*p*	r
Decayed Teeth	4.28 *	3.20	2.98	2.59	0.048	0.223
Missing Teeth	2.21	2.17	1.60	1.46	0.369	0.101
Filled Teeth	8.13	5.30	6.58	3.57	0.241	0.132
Decay-Missing-Filled Teeth	14.62 ^#^	5.71	11.15	4.15	0.004	0.326
Dental Treatment Index	0.62	0.31	0.66	0.27	0.609	0.058
CARIES SEVERITY	0.37	0.15	0.36	0.15	−0.677	0.498

Legend: * *p* < 0.05 # *p* = 0.004.

**Table 3:** The levels of selected biochemical and physicochemical salivary parameters in the cystic fibrosis (CF) and the control group.

	CF Group (*n* = 40)		Control Group (*n* = 40)		
	M	SD	M	SD	*p*
**total sialic acid (TSA) (mg/L)**	65.19	6.42	42.66	4.45	<0.001
**total protein (mg/mL)**	5.06	21.03	1.16	0.21	<0.001
**lysozyme levels (μg/mL)**	0.40	0.37	0.50	0.10	<0.001
**lactoferrin levels (μg/mL)**	3.41	0.84	1.93	0.68	<0.001
**α-amylase activity (U/mL)**	91.03	12.10	55.74	9.00	<0.001
**resistin levels (ng/mL)**	6.34	0.77	7.88	0.77	<0.001
**TNF-α levels (pg/mL)**	21.96	2.85	8.74	4.33	<0.001
**buffer capacity**	6.04	0.52	6.73	0.21	<0.001
**pH**	6.74	0.60	7.07	0.14	0.012

**Table 4:** An analysis of correlations between the levels of selected salivary parameters and total number of decayed, filled and missing teeth (DMF) and Dental Treatment Index (DTI) in the control group.

Correlation	*r*	*p*
Lactoferrin levels vs. F	−0.351	0.026
Lactoferrin levels vs. DMF	−0.399	0.011
α-amylase activity vs. F	0.349	0.027
α-amylase activity vs. DMF	0.390	0.013
pH vs. DMF	−0.342	0.031
Salivary buffer capacity vs. DMF	−0.326	0.040
Total protein vs. DMF	−0.393	0.012

F-filled teeth, DMF-decayed, missing and filled teeth.
